# Novel Application of Statistical Methods to Identify New Urinary Incontinence Risk Factors

**DOI:** 10.1155/2012/276501

**Published:** 2012-10-31

**Authors:** Theophilus O. Ogunyemi, Mohammad-Reza Siadat, Suzan Arslanturk, Kim A. Killinger, Ananias C. Diokno

**Affiliations:** ^1^Department of Mathematics and Statistics, Oakland University, 2200 N. Squirrel Road, Rochester, MI 48309, USA; ^2^Department of Computer Science and Engineering, Oakland University, Rochester, MI 48309, USA; ^3^Beaumont Health System, William Beaumont Hospital, Royal Oak, MI 48073, USA; ^4^Oakland University William Beaumont School of Medicine, Rochester, MI 48309, USA

## Abstract

Longitudinal data for studying urinary incontinence (UI) risk factors are rare. Data from one study, the hallmark Medical, Epidemiological, and Social Aspects of Aging (MESA), have been analyzed in the past; however, repeated measures analyses that are crucial for analyzing longitudinal data have not been applied. We tested a novel application of statistical methods to identify UI risk factors in older women. MESA data were collected at baseline and yearly from a sample of 1955 men and women in the community. Only women responding to the 762 baseline and 559 follow-up questions at one year in each respective survey were examined. To test their utility in mining large data sets, and as a preliminary step to creating a predictive index for developing UI, logistic regression, generalized estimating equations (GEEs), and proportional hazard regression (PHREG) methods were used on the existing MESA data. The GEE and PHREG combination identified 15 significant risk factors associated with developing UI out of which six of them, namely, urinary frequency, urgency, any urine loss, urine loss after emptying, subject's anticipation, and doctor's proactivity, are found most highly significant by both methods. These six factors are potential candidates for constructing a future UI predictive index.

## 1. Introduction

The Medical, Epidemiologic, and Social aspects of Aging (MESA) project, funded by the National Institutes on Aging (NIA), was a multistage longitudinal observational population-based study that began in 1983 and focused on factors related to the epidemiology of urinary incontinence (UI). Investigators conducted repeated and detailed household and telephone interviews (four interviews at approximately 1-2 year intervals) of 1955 seniors, 60 years and older, drawn from a probability sample of 13912 households in Michigan [1]. The enormous volume of urinary and health-related data that were collected is now contained in the MESA database. These data have significantly contributed to the study of the prevalence, incidence, and factors related to UI as well as diagnostic methods and initiatives to prevent UI in the elderly [1–3]. 

MESA data have been examined; however, detailed statistical modeling methods that are crucial for examining transitions over time have not been applied to examine change patterns and risk factors associated with developing UI. Although both men and women experience UI, the prevalence in women is almost double that of men [1]. More than one in three adult women in the United States suffers from UI [4]. One meta-analysis reported ranges of UI prevalence from 4.5% to 44% (mean 23.5%) in healthy women and 4.6% to 24% (mean 14.5%) in men 60 years or older [5]. 

In light of the size of the MESA data and possible complications often posed by missing values and possible multicolinearity among some attributes, we used a novel application of statistical methodologies to examine the volumes of data contained in the MESA database. Evaluating these methods tests their utility in mining MESA and other large data sets and is our first step towards developing a predictive UI index. Developing a clinically useful index to predict the risk of developing UI based on longitudinal observations of female MESA subjects would provide an evidence-based approach to identifying those that would benefit most from early and aggressive prevention strategies such as behavioral modification. We were also interested in comparing the performance of the GEE and PHREG methods in identifying significant risk factors from the MESA data to the logistic regression methods which had been previously applied only to the baseline data [6]. The purpose of this paper is to report possibly new risk factors associated with becoming incontinent using a novel statistical data mining methodology on MESA data as a preliminary step to creating a clinically useful predictive index for UI. 

## 2. Materials and Methods

### 2.1. Study Design

This was a longitudinal observational study.

### 2.2. Data Collection

The details of the MESA studies had been previously described [1]. Institutional Review Board approval was obtained prior to conducting the current analysis of MESA data. Briefly, a multi-stage probability sample of 13.912 households in Washtenaw County, Michigan, was developed. Senior citizens 60 years or older living in the sampled households (*n* = 3.005) were invited to participate. A total of 1.955 community-dwelling seniors (1.099 subjects age 60–69 years, 589 age 70–79 years, and 268 age 80 and up; 59% women; 91% white) were interviewed in their homes for approximately 2 hours at baseline (1983-1984 interview) and then reinterviewed at 1-2-year intervals. Trained interviewers collected the data in a consistent manner with standardized language and explanations throughout. Repeated and scripted in-person and phone interview techniques enabled verification, validation, and additional probing of respondents' answers. Reinterview response rates ranged from 69% to 72% in those subjects that were still living [7]. Respondents, or their living relatives, were contacted a fourth time in 1990 to explore the role of UI as a risk factor for mortality [8]. For the current analysis, 702 continent women at baseline that also had 1 year data were identified. The mean age was 69 years, and 93.2% were white, 6.5% African American, and 0.3% “other” race. Of these, 82 became incontinent at 1 year. The current study focuses only on the baseline and first follow-up interview 1-2 years later.

MESA investigators defined incontinence as a loss of any urine volume with a minimum frequency of 6 days within the last 12 months, and this definition of incontinence was also used in the present analysis. Subjects determined to be incontinent at 1 year follow up were further questioned to determine characteristics and precipitating factors of urine loss. Medical history, mobility, cognitive function, current health, and quality of life data were also collected. There were 762 survey questions in the baseline survey and 559 questions in the follow-up survey.

### 2.3. Statistical Analysis

Since the current study focuses on identifying new significant risk factors for incontinence by employing two methods that exploit possible correlation between the observed values of the urinary incontinence response variable at the baseline and followup, we only analyzed the data provided by female MESA participants that had both baseline and 1 year data (702 cases). 

Women who were already classified as incontinent (6 or more days of urine leakage within the past 12 months) at the baseline, but were undergoing treatment at that time, were not included in this study. Of the 73 factors common to both the baseline and follow-up data, only about 5% of the seventy-three have 10%–48% missing values (for the baseline) and 10% have 15%–52% missing values (for the followup). We imputed values for the missing values using regression, logistic regression, or MCMC method under the SAS MI procedure, depending on whether the factor is continuous or categorical and whether the missing pattern is monotone or arbitrary. But before we carried out the imputation, we eliminated 35 out of the 702 cases that are over 50% missing data.  Table 1 summarizes the percentages of missing values in both the baseline and follow-up data sets. The issue of the skip pattern factors is nontrivial to handle because they appear as missing values when combined along with the nonskip pattern factors, but they are not truly missing. Ten of the skip pattern questions that were also repeated in the follow-up questionnaire and with at least 75% response rates were also included among the 73 common risk factors.

 Most of the variables used in the MESA data analyses were binary, and the ones that were not binary were dichotomized, similar to the Harvard Report on Cancer Prevention [9] which also involved a large number of factors. Sixty of the 73 factors used were categorical and many of them had several levels, and the remaining 10 factors were quantitative. With ten quantitative factors, it would have been an herculean task to run an analysis of covariance. For most of the factors that were dichotomized, we relied on experts' opinions, and in four cases, we used the distributions of the values of factors to make judgments. In the case of possible colinearity, we did not find cases of severe multicolinearity (applying the variance inflation factor of 10 or higher) among the common 73 risk factors we used in the analysis. 

All the analyses used statistical procedures from SAS [10]. We applied the SAS Genmod Procedure [10] to the baseline and one year follow-up data, based on the generalized estimating equation (GEE) [10] method to exploit the nature of the repeated measurements contained in the data. In light of the size of the MESA data, complications often posed by missing values, and possible colinearity among some attributes (though we did not find any significant level of colinearity among the 73 factors), an alternative method, called proportional hazard regression (PHREG) [10], was also applied to the data to strengthen the analysis. The results from the two methods are very close, and they are exhibited in Table 2. On the other hand, we used the SAS Logistic procedure [10], which had been used in previous studies on the baseline data, to identify significant factors from the baseline data. The baseline data used in this case consist of the twenty-eight of the baseline questions that were not repeated during the follow-up survey. The results from the logistic regression are listed in Table 3.

 A further analysis of the risk factors listed in Table 2, identified by the GEE and PHREG methods and F16 and F17 identified by the logistic regression from Table 3, was carried out according to the types of urinary incontinence, namely, stress and overflow incontinence, as well as using other criteria. Also we were able to incorporate interactions among the risk factors into this analysis. Since F16 and F17 were only from the baseline data, we used only the baseline data set in this further analysis. We still applied dichotomization to the nonbinary factors into the binary type, as listed in Table 2. This time, we applied the method of weighted analysis of variance to categorical data available in SAS Catmod Procedure [10]. The method of weighted least squares allowed us to model directly the proportion of cases with urinary incontinence in each classification group. Using the proportion as a response function has the interpretative advantage that model parameters have a direct effect on the size of the proportions. We started fitting a saturated model containing all of the main and interaction effects (that SAS and data allowed) and ended up with a reduced model under each urinary incontinence type. The result of the analysis under each type of incontinence is displayed in Table 4. Only the results of a final reduced model under each case of incontinence type are displayed. Each of the final reduced models is a good fit to the data, with at least a *P* value of 0.10. 

## 3. Results 

 We investigated identification of possible new significant risk factors for urinary incontinence from the baseline and follow-up MESA data over a period of one year. The risk factors that were found significant by the GEE and PHREG methods, based on the repeated data, are listed in Table 2. For the PHREG result, we also included the hazard ratio. If the hazard ratio of a factor is larger than 1, an increment in the factor increases the hazard towards incontinency, and if it is less than 1, an increment in the factor decreases the hazard towards incontinence. Both methods essentially found the same set of 15 significant factors, except for factors F5 and F11 where the two methods did not quite agree. For the 28 baseline variables that were not repeated in the follow-up survey, F16 and F17 were found significant by the logistic regression model. They are listed in Table 3. It is very important to state that there are six risk factors that are found highly significant by both methods, namely, F4, F6, F7, F8, F10, and F15 that deal with urinary frequency, urgency, any urine loss, urine loss after emptying, subject's anticipation, and doctor's proactivity, respectively. These six risk factors, along with others, will certainly be good candidates for constructing a future prediction index for urinary incontinence in older women. According to the literature, the following factors that GEE and PHREG methods found significant have not been reported in any previous study based only on MESA baseline data: F1, F2, F8, F9, F10, F12, F13, F14, and F15. 

 The risk factors listed in Table 2 and F16 and F17 in Table 3 were further categorized as associated with stress, urge or overflow incontinence, disease, anticipatory care, or hereditary factors, and analyzed (Table 4). The final reduced model for the stress group consists of risk factors F11 (amount of weekly exercise) and F17 (sneezing frequency) that were found significant (Table 4). The estimate of the parameter for F11, 0.0518, being positive means doing a little or no exercise at all may be symptomatic of tendency towards incontinence. The case of F17 (sneezing) is similar in the sense that sneezing often or sometimes may be symptomatic of tendency towards incontinence. There is no significant interaction. For the subjects with a little or no exercise at all, the predicted proportion with the stress incontinence symptom is 0.405 ± 0.026 while that of the subjects who sneezed often or sometimes is 0.437 ± 0.047. The other risk factors considered in this stress incontinence group, F12 (errands' frequency) and F18 (coughing frequency), are found to be nonsignificant. 

 For the urge incontinence group, we consider the risk factors F6 (trouble getting to the bathroom on time) and F7 (frequency of wetting or soiling self). The results of their reduced final model are shown in Table 4. Their interaction is not significant. The parameter estimate, 0.0812 for F6, being positive means having trouble getting to the bathroom on time increases the chance of experiencing urge incontinence, while the parameter estimate, 0.2791, for F7 indicates that wetting or soiling oneself at least once a week is obviously associated with urge incontinence. The predicted proportions of subjects with urge incontinence with regard to F6 and F7 are 0.351 ± 0.045 and 0.747 ± 0.042, respectively. 

 The risk factors considered under the overflow case are F4 (voiding frequency in 24 hours) and F8 (occasional leakage after voiding). Their interaction is not significant, and their parameters' estimates are 0.0727 and 0.2392, respectively, meaning voiding more than 8 times on average a day or occasionally experiencing wet undergarments or clothing may be associated with developing the MESA definition of incontinence (urinary leakage on at least 6 days per year). The respective predicted proportions for F4 and F8 are 0.417 ± 0.031 and 0.750 ± 0.044, respectively.

 The disease group consisting of F3 (blood, cloudiness, or smell in urine), F14 (high blood pressure), and F9 (memory loss) were all found significant, but with no significant 2-factor or 3-factor interactions. With all their parameters' estimates being positive, (0.156, 0.020, and 0.050, resp.), each of the three factors increases the chance of experiencing urinary incontinence. Their respective predicted proportions are 0.427 ± 0.031, 0.700 ± 0.063, and 0.640 ± 0.064.

 The anticipatory group consists of the risk factors F10 (possible future urinary incontinence) and F15 (doctor's proactive investigation). These were the only two risk factors with a significant interaction that we found. The parameter estimate for their interaction is 0.0729, which indicates an increase in the chance towards urinary incontinence if a subject does not anticipate developing such a problem and if there is a nonproactive approach on the part of a doctor in probing a possibility of such a development of urinary problem. To our knowledge, these potential risk factors have not been previously reported in the literature. Both GEE and PHREG methods found them significant, based on the baseline and follow-up data, and the analysis of weighted least squares, based only on the baseline data, also found them significant.

The last group to consider is the hereditary factor, F16, group. In light of the preceding discussion, we wanted to combine F10, F15, and F16, but there were not enough data to cover all the eight classifications they jointly generate. Again, the positive value of the parameter estimate for F16 indicates the possibility that if one of the subject's parents had urinary incontinence, the subject's chance of developing UI is increased with the chance being about 35.6%  ± 2.1%, based on the predicted proportion. As usual and as in many diseases, this calls for a subject's awareness of family medical history.

It is very important to note that there are six risk factors that were found to be highly significant by both methods, namely, F4, F6, F7, F8, F10, and F15 reflecting urinary frequency, urgency, minor urine loss or urine loss after emptying that does not meet the MESA definition of incontinence, subject's anticipation, and doctor's proactivity, respectively. These six risk factors, along with perhaps others, are promising candidates for constructing a future prediction index for urinary incontinence in older women. 

## 4. Discussion

MESA investigators have reported many of their findings, yet many of these robust data have not been fully examined. Previous MESA studies have also been more descriptive in nature and did not employ the statistical methods for repeated data required for identifying risk factors over two periods (baseline and followup). Diokno et al. reported relationships between incontinence and a wide range of chronic conditions, symptoms, and treatments [6] but did not take advantage of the repeated measures nature of MESA data. In an unpublished study, investigators applied the stepwise (backward) procedure under a logistic regression model to identify risk factors, but only to the MESA baseline data. In the current study, we applied the GEE and PHREG methods to the baseline and follow-up data sets, using repeated measures analytical tools. To the best of our knowledge, this is the first study of its kind that analyzes the MESA data in a detailed two-period repeated measures method with the goal of identifying new potential risk factors associated with developing incontinence. The two competing methods applied, GEE and PHREG, substantively identified the same set of significant risk factors from the two-period longitudinal analysis. 

 Two key issues to address in data preparation for analysis from any survey are the level of missing values and amount of skip pattern questions. We applied imputation methods to take care of the missing values. However, with regard to the skip patterns, it would be grossly misleading to wrongly classify a nonresponse as missing when the non-response may be part of a skip pattern. A skip pattern will jump a respondent over a group of questions that is not relevant to him or her. Because missing skip pattern data are unlike the missing data from questions where a response would always be expected, we only included ten of these skip pattern attributes where it was reasonable to do so. Including many skip pattern variables would generate unmanageable amounts of missing values, and blending many of them with non-skip pattern attributes in the same analysis would cause serious computational problems and misleading conclusions. However, a detailed study of the analysis of skip pattern factors is currently being conducted, and findings will be incorporated into our development of a predictive incontinence index in the future. 

The most illuminating and, to our knowledge, newly identified UI risk factor is the highly significant finding of F15 by both methods: “When you go for visits to your doctor (regular doctor), does your doctor ask you if you are having any problems with urine loss or bladder control problems?” The high significance level of this factor might indicate that proactive physician assessment appears to play a significant role in reducing the risk of developing incontinence. The relationship between patients' anticipation of developing urinary incontinence, proactive UI assessment by physicians, and eventually developing UI has not been previously reported. In our study, both GEE and PHREG methods found these factors significant, and the analysis of weighted least squares also found them significant. Subjects' awareness of a potential UI problem (as asked in F10) and a doctor's proactive approach to such a problem (as asked in F15) form a powerful combination and may be extremely important clinically in the prevention of UI or even other conditions. 

This finding may signify several important implications. First, public health education efforts might focus on increasing UI awareness. Secondly, health care providers might be instructed on the importance of proactive UI assessment. Lastly, the efforts of Physician Quality Reporting System should be applauded for its voluntary initiative to improve UI assessment. This initiative provides physician incentives for UI assessment in women ≥65 years old, documentation of UI characteristics, and developing a plan of care. Although voluntary programs such as these may improve UI prevention, the current study suggests or confirms that perhaps mandatory UI assessment may be of benefit. The aging patient who has not considered that they might develop UI might not be in tune to small urine leaks or subtle increases in urinary urgency. These new findings arising from MESA data are highly insightful and suggest that probing condition-specific questions will promote early identification, intervention, and perhaps even prevention. This interesting finding warrants more study to not only confirm these results but to also evaluate the practicality of incorporating routine screening into practice, if it is not already in place.

There are some limitations to this study. For example, we were not able to include some factors such as sneezing, coughing, pregnancy, or number of births into the GEE and PHREG models since these and other factors were only asked during the baseline interview. Analyzing a large number of factors that have more than two levels of responses can also be challenging and can lead to sparsity of data. We experienced such a scenario in our analysis, which led to the dichotomizing variables with multiple response levels previously discussed. Also, based on experts' opinions, we dichotomized the nonbinary factors. Given the number of variables that had multiple levels and/or were quantitative, it would have been an herculean task to run an analysis of covariance. 

To date, no UI predictive index has been developed or tested for widespread use in women. Although Burgio et al. identified several risk factors associated with postpartum incontinence [11], no predictive index was established. Furthermore, most previous studies of UI risk factors have been derived from cross-sectional studies of volunteers and clinical subjects [12], and UI definitions varied, which limits their usefulness for UI prediction in the general population. Since many seniors suffer from UI, there is great potential to improve health and quality of life by developing a UI predictive index for this population. UI is underreported and undertreated [13], contributes to social isolation, depression, and dependency, and is a significant factor in nursing home admissions [14]. The economic burden of UI is greater than the combined direct cost for breast, cervical, and ovarian cancers [15] and imposes significant burden on individuals, families, and communities.

## 5. Conclusions

Our data mining efforts have confirmed not only some previously identified risk factors, but also associations between risk factors that will be useful in our efforts to construct a UI predictive index. Furthermore, new findings that may influence prevalence of urinary incontinence, namely, patients' anticipation of becoming incontinent and doctors' proactive assessment of incontinence, were revealed using a repeated design model based on GEE method and PHREG model which reinforced the analysis. Utilizing this double-barrel statistical model on a two-period longitudinal analysis has never been carried out on MESA data and is a more informative and appropriate approach to identify key risk factors involved in developing incontinence. To construct a clinically useful index to predict UI in women, indepth applications of mathematics, statistics, and simulation will be needed. Since the percentage of women over 60 years old is continually growing as average life expectancy increases, the negative implications of UI are likely to increase as well. Using a scientifically developed and tested predictive UI index will more readily identify at-risk women and permit widespread prevention or early treatment. 

## Figures and Tables

**Table 1:** Percentages among 73 common attributes in the baseline and follow-up data with missing values.

	Percent of attributes with the range of missing values
Baseline data	
0%–9%	95%
10%–48%	5%
Follow-up data	
0%–14%	90%
15%–52%	10%

**Table 2:** Significant risk factors identified by the GEE and/or PHREG methods.

	Questions	Possible responses	Response associated with higher incontinence risk	GEE*	PHREG**	
				*P* value	*P* value	Hazard ratio
F1	What about problems with constipation in the past 12 months?	(1) Yes (2) No (3) Don't know.	Yes	0.0108	0.0162	1.53
F2	In general, how would you describe your health? Would you say that it is excellent, very good, good, fair, or poor?	(1) Excel (2) V. Good (3) Good (4) Fair (5) Poor (6) Don't know	Poor	0.0139	0.0122	0.57
F3	Have you had any blood, cloudiness, or foul smell in your urine in the past 12 months?	(1) Yes (2) No (3) Don't know.	Yes	0.0287	0.0241	2.02
F4	In general, over a 24-hour period, about how many times do you go to the toilet and urinate?		At least 8 times	0.0038	0.0026	1.60
F5	Have you ever had female surgery such as your ovaries, vagina, fallopian tubes, uterus, rectum, or urethra?	(1) Yes (2) No (3) Don't know.	Yes	0.0044	0.0620	1.70
F6	Do you have trouble getting to the bathroom on time?	(1) Yes (2) No (3) Don't know.	Yes	<0.0001	<0.0001	6.86
F7	How often do you wet or soil yourself during the day or night?	(1) Would you say never (2) Less than once a week (3) Once or twice a week (4) Three or more times a week	At least once a week	<0.0001	<0.0001	31.53
F8	Some people find that occasionally they experience wet undergarments soon after they empty their bladders into the toilet. In other words, the bladder feels empty, but later leaks more. Is this ever true for you?	(1) Yes (2) No (3) Don't know	Yes	<0.0001	<0.0001	4.75
F9	How about your memory? Has it become worse within the last five years?	(1) Yes (2) No (3) Don't know.	Yes	0.0224	0.0141	1.47
F10	How likely do you think it is that you will have a urine loss condition in the future?	(1) Is it very likely (2) Somewhat likely (3) A little likely (4) Not likely at all	(1) Very likely (2) Somewhat likely	0.0003	<0.0001	3.34
F11	In an average week, would you say that you get a large amount of exercise, a fair amount of exercise, a little exercise, or no exercise?	(1) Large amount, (2) Fair amount (3) A little (4) No exercise at all (5) Don't know	(3) A little (4) No exercise at all	0.0098	0.0563	1.44
F12	How often do you run errands outside the home for either family members or friends? (Would you say…)	(1) Al. everyday (2) ≥1/week (3) 1 or 2/month (4) Few/year (5) Less often than that (6)Don't know.	(4) Few/year, or (5) Less often than that	0.0333	0.0236	0.69
F13	In the past 12 months, have you had to use a catheter or urine tube to empty your bladder?	(1) Yes (2) No (3) Don't know	Yes	0.0133	0.0038	0.20
F14	Have you ever been told by a doctor that you had high blood pressure?	(1) Yes (2) No (3) Don't know.	Yes	0.0287	0.0148	0.66
F15	When you go for visits to your doctor (regular doctor), does your doctor ask you if you are having any problems with urine loss or bladder control problems?	(1) Yes (2) No (3) Not regular (4) Don't know	(2) No or (3) Not regularly	0.0062	0.0028	0.62

*GEE: generalized estimating equation.

**PHREG: proportional hazard regression model.

**Table 3:** Baseline risk factors found significant by logistic regression.

	Questions	Possible responses	Response associated with higher incontinence risk	*P* value
F16	Did your mother or your father have a urine loss condition as an adult?	(1) Yes (2) No (3) Don't know.	Yes	0.0135
F17	Do you sneeze often, sometimes, rarely, or never?	(1) Often (2) Sometimes (3) Rarely (4) Never (5) Don't know	(1) Often	0.0171
F18	Do you cough often, sometimes, rarely, or never?	(1) Often (2) Sometimes (3) Rarely (4) Never (5) Don't know	(1) Often	0.0972*

*Not significant at 5% significance level, but it is included for a future closer look.

**Table:** (a) Stress urinary incontinence

Factor	Estimate	SE	Chi-Square	*P* value
F17	0.0535	0.0237	5.09	0.0241
F11	0.0470	0.0237	3.93	0.0475

**Table:** (b) Predicated values for response functions

	Observed function	SE	Predicted function	SE
F17	0.394822	0.027808	0.394822	0.027808
F11	0.381818	0.06551	0.381818	0.06551

**Table:** (c) Urge urinary incontinence

Factor	Estimate	SE	Chi-square	*P* value
F6	0.0812	0.0221	13.49	0.0002
F7	0.2791	0.0215	168.37	<0.0001

**Table:** (d) Predicated values for response functions

	Observed function	SE	Predicted function	SE
F6	0.433962	0.068079	0.351143	0.044893
F7	0.791045	0.049669	0.74696	0.041532

**Table:** (e) Overflow urinary incontinence

Factor	Estimate	SE	Chi-square	*P* value
F4	0.0727	0.0182	15.89	<0.0001
F8	0.2392	0.0219	119.32	<0.0001

**Table:** (f) Predicated values for response functions

	Observed function	SE	Predicted function	SE
F4	0.43128	0.034095	0.416656	0.031473
F8	0.8	0.063246	0.749681	0.044325

**Table:** (g) Disease

Factor	Estimate	SE	Chi-square	*P* value
F14	0.0197	0.0184	1.14	0.2857
F3	0.1558	0.0304	26.34	<0.0001
F9	0.0498	0.0185	7.22	0.0072

**Table:** (h) Predicated values for response functions

	Observed function	SE	Predicted function	SE
F14	0.8	0.126491	0.639677	0.63708
F3	0.456044	0.036919	0.427493	0.031484
F9	0.555556	0.117121	0.699794	0.062957

**Table:** (i) Anticipatory factors

Factor	Estimate	SE	Chi-Square	*P* value
F15	0.1008	0.0255	15.55	<0.0001
F10	0.1917	0.0255	56.29	<0.0001
F10 × F15	0.0729	0.0255	8.14	0.0043

**Table:** (j) Predicated values for response functions

	Observed function	SE	Predicted function	SE
F15	0.347578	0.025418	0.347578	0.025418
F10	0.529412	0.085601	0.529412	0.085601
F10 × F15	0.291866	0.031447	0.291866	0.031447

**Table:** (k) Hereditary factors

Factor	Estimate	SE	Chi-square	*P* value
F16	0.0791	0.0218	13.11	0.0003

**Table:** (l) Predicated values for response functions

	Observed function	SE	Predicated function	SE
F16	0.356275	0.021547	0.356275	0.021547
